# Relationship between the geometry patterns of vertebrobasilar artery and atherosclerosis

**DOI:** 10.1186/s12883-018-1084-6

**Published:** 2018-06-12

**Authors:** Jin Yu, Shu Zhang, Ming-Li Li, Yi Ma, Yu-Ru Dong, Min Lou, Feng Feng, Shan Gao, Shi-Wen Wu, Wei-Hai Xu

**Affiliations:** 1 Department of Neurology and Radiology, General Hospital of Chinese People’s Armed Police Forces, 69 Yongding road, Haidian District, Beijing, 100039 China; 2 Department of Neurology and Radiology, Peking Union Medical College Hospital, Chinese Academy of Medical Sciences, Shuaifuyuan 1, Dongcheng District, Beijing, 100730 China; 3 Department of Neurology, Zhejiang University 2nd Affiliate Hospital, Hangzhou, Zhejiang Province China

**Keywords:** Intracranial atherosclerosis, Vertebrobasilar system, Geometry, Plaque, Magnetic resonance imaging

## Abstract

**Background:**

The plaques at the dorsal or lateral wall of basilar artery (BA) are associated with pontine infarcts. We sought to explore the correlations between vertebrobasilar artery geometry and BA plaque locations.

**Methods:**

We retrospectively analyzed the imaging and clinical data of 84 patients with BA atherosclerosis. On three-dimensional time-of-flight images, a side to side diameter difference of bilateral vertebral artery (VA) and BA bending were assessed. The vertebrobasilar artery geometry was qualitatively classified into four basic configurations: Walking, Tuning Fork, Dominant-Lambda, and Hypoplasia-Lambda. On high-resolution magnetic resonance imaging, the plaques were categorized based on the involvement of the ventral, dorsal, or lateral sides of BA wall. The relationships between vertebrobasilar artery geometry parameters and plaque locations were analyzed.

**Results:**

Left VA dominance was identified in 28(33%) patients, and right VA dominance in 22(26%) patients. BA bending were detected in 49 patients. There were no significant correlations between the diameter difference/ratio of VA diameters and plaque locations, or between BA bending and plaque locations. BA plaques were evenly distributed in the vertebrobasilar arteries with Tuning Fork and Dominant-Lambda configurations. In Hypoplasia-Lambda group, however, plaques were more frequently located at the dorsal wall (58.57%) than at the ventral (14.43%) and lateral wall (26.71%; *P* = 0.001). In Walking group, the plaques more likely occurred at the lateral (49.79%) and dorsal (35.07%) wall than at the ventral wall (14.86%, *P* = 0.02).

**Conclusions:**

The geometric configurations of vertebrobasilar artery strongly influence the BA plaque locations. Further prospective studies are warranted to testify whether Hypoplasia-Lambda and Walking configurations are independent risk factors for pontine infarcts.

**Electronic supplementary material:**

The online version of this article (10.1186/s12883-018-1084-6) contains supplementary material, which is available to authorized users.

## Background

Vertebrobasilar atherosclerosis is a common cause of ischemic stroke. The underlying mechanisms include artery-to-artery embolism, in situ thrombo-occlusion, local branch occlusion, or hemodynamic impairment [1]. Local branch occlusion is the relatively more important mechanism, because the small and short perforating vessels of basilar artery (BA) are vulnerable to occlusion in the presence of parental artery atherothrombosis [1]. Recent in vivo studies have provided supportive evidence that the plaques at the dorsal or lateral wall of BA, where the branch arteries arise from, are associated with symptomatic pontine infarcts [2, 3].

It is known that mechanical and hemodynamic factors play a role in the development of atherosclerosis [4]. Early atherosclerotic plaques often develop at the sites with low or oscillatory wall shear stress [5], such as the inner wall of a curved artery, the outer wall of a bifurcation, and the apex of a junction. Unlike other systemic arteries with a tree-like branching pattern, the BA is the only artery in humans in which two flows merge. The diameters of the vertebral artery (VA) are of equal size in 6–38.5% of patients in angiographic or postmortem studies [6, 7]. The variations of the geometry and asymmetrical inflow of vessels result in changes of the flow force distribution, that influenced the morphological deformation in the vertebrobasilar system (VBS) and induced infarcts in the different areas [8]. We hypothesized certain VBS geometry may be associate with certain distribution of plaque, which may influence the etiologies of infarction. In this study, using high-resolution magnetic resonance imaging (HR-MRI), we analyzed the relevance of the VBS geometry and BA plaque locations.

## Methods

### Patients

We retrospectively reviewed the HR-MRI databases (2008 to 2015) from three medical centers. All patients with BA atherosclerotic plaque on HR-MRI were enrolled if they fulfilled the following criteria: (1) had two normal VAs detected by magnetic resonance angiography (MRA); (2) image quality good enough for analysis. This observational study was approved by the local ethics committees. All the subjects signed an informed consent.

### Image analysis

Details of our high-resolution MRI protocol were described elsewhere [3]. The images of BA wall were graded on a 3-point scale: 1 = nonvisualization, 2 = adequate, and 3 = good. The images with a score of 2 or 3 were analyzed [8]. On MRA, the diameter of each VA was calculated as the average of the measurements made at three consecutive points, 3 mm apart, starting from the vertebrobasilar junction [9]. The dominant VA was defined if it was larger in diameter (difference ≥ 0.3 mm) than the contralateral side [6]. The ratio of VA diameters was defined according the following formula: ratio = one side of diameter/the other side of larger diameter. A line was drawn between the top of the BA and junction of both VAs for reference to decide the side of BA bending [10].

**Fig. 1:**
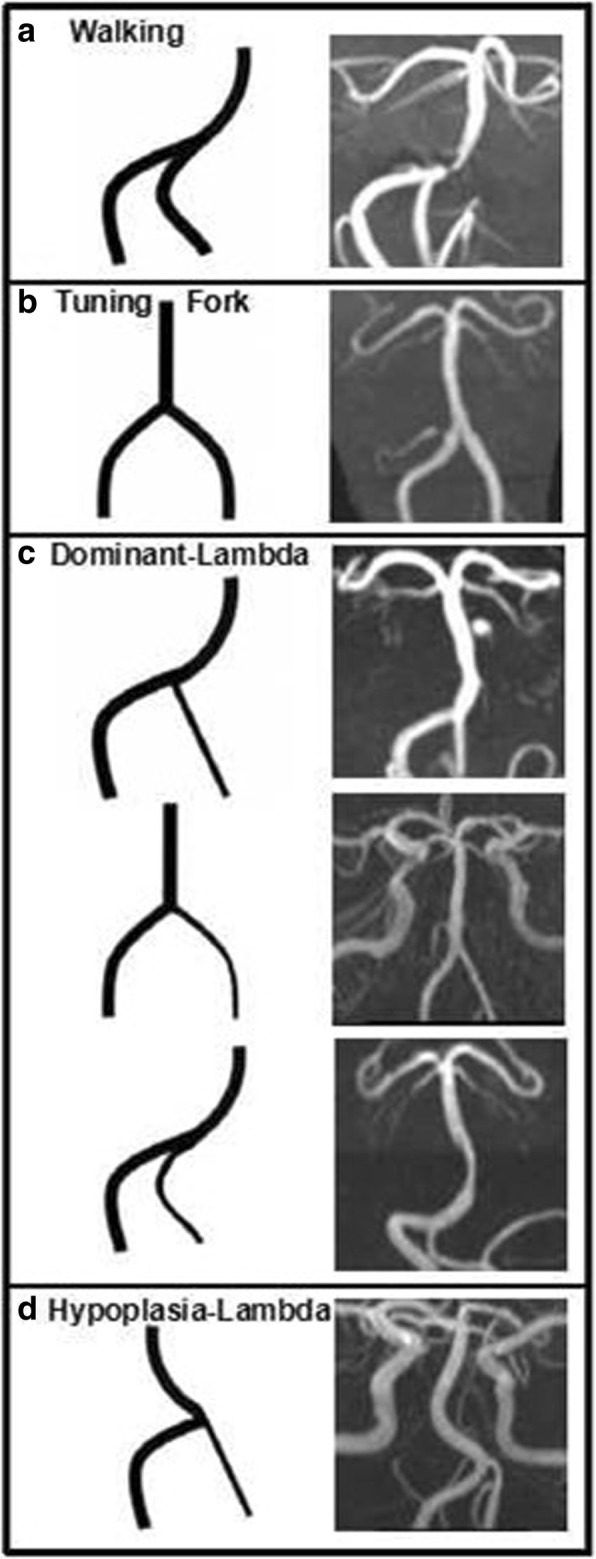
Vertebrobasilar configurations. The vertebrobasilar system geometry is qualitatively classified into four basic geometric configurations: Walking (**a**), Tuning Fork (**b**), Dominant-Lambda (**c**), and Hypoplasia-Lambda (**d**). In each panel, a schematic of the configuration is followed by anterior-posterior magnetic resonance angiography

Based on the TOF images, the VBS geometry was qualitatively classified into four basic geometric configurations: Walking, Tuning Fork, Dominant-Lambda, and Hypoplasia-Lambda (Fig. 1) [11]. The Walking geometry is distinguished by two equal VAs (diameter difference <0.3 mm) that bend in the same direction before merging into the BA [11]. The Tuning Fork configuration exhibits the two equal VAs that join at the confluence at rather symmetrical angles with respect to the BA, and the VAs bend in opposite directions [11]. In the Dominant-Lambda configuration, the BA path follows the direction dictated by the dominant VA, and the other VA, typically smaller, abuts the dominant VA or BA path, forming a pseudo T-junction [11]. In the Hypoplasia-Lambda configuration, the BA path follows the direction dictated by relatively hypoplastic VA.

Details of the design of plaque distribution have been published previously [3]. Briefly, all cross-sections with eccentric plaque were classified based on their plaque orientation being centered on the ventral, dorsal, and lateral (left or right) sides of the vessel. All images were reviewed by two experienced readers (Yu and Xu) blinded to clinical data, using software of the syngofast View-Viewer for DICOM images (Ver.1.0.0.34). The differences between the two observers were solved by consensus. VA diameters in the initial 10 patients were remeasured 2 months later for estimation of intraobserver and interobserver variability.

### Statistical analysis

Intraobserver or interobserver variability for the measurements of VA was determined by intraclass correlation coefficient (ICC). Quantitative data are expressed as mean ± SD and qualitative data are expressed as percentage. The continuous variables between the two groups were compared by the independent samples t-test. For each stenosis, the percentage of individual plaque distribution was calculated (Additional file 1: Table S1). The mean ventral, dorsal, lateral (left and right) plaque orientation of the total group was derived from the individual percentage distribution [12]. The comparison of the plaque distribution among different walls was performed by a Kruskal-Wallis test of the mean percentage of the distribution for each individual stenosis followed by Variance analysis and Bonferroni correction for multiple comparisons. Data comparisons among the four groups of vessels were conducted with the Wilcoxon test. Multiple correlation analysis between the ratio/difference of VA diameters and the percentage of individual plaque distribution from each patient were calculated. Correlation between the plaque distribution and the side of bending of the BA were assessed by multiple logistic regression analysis. A probability value of <0.05 was considered statistically significant.

## Results

One hundred and nineteen patients with BA plaque were considered for enrollment. Five patients with poor image qualities and thirty patients with stenotic VAs were excluded. Eighty-four (mean age 62.4 ± 12.9 years, 61 male) patients were finally included for analysis. Forty-six patients (54.8%) were symptomatic and new ischemic lesions were identified on diffusion-weighted imaging. Of the symptomatic patients, 29 had pontine infarctions, 1 had infarctions involving both pontine and extra-pontine area (with a cerebellum infarction), and 9 with extra-pontine infarctions (3 with thalamic infarction, 2 with occipital lobe infarction, 1 with cerebellum infarction, and 1 with medulla oblongata infarction). Eighteen patients (21.4%) with BA atherosclerotic stenosis (≥50%) were detected by MRA. There were 34(40.5%) patients with hypertension, 29(34.5%) with hyperlipidemia, 20(23.8%) with diabetes mellitus, and 20(23.8%) smoking patients.

The intraobserver variability was small for the measurements of VA (ICC 0.995, 95% CI 0.980–0.999). The interobserver variability was also small for the measurements of VA (ICC 0.993, 95% CI 0.972–0.998).

The average diameter of VA was 2.45 ± 0.68 mm in the left and 2.35 ± 0.52 mm in the right (*p* = 0.17). The diameters of the VA were of equal size in 34(40.5%)patients. Of the remaining 50 patients, the left VA dominance was identified in 28 (56%) patients and the right VA dominance in 22 patients (44%). The difference of VA diameters ranged from 0.0 to 1.8 mm, and the ratio from 0.4 to 1.0. There were no significant correlations between the difference/ratio of VA diameters and the plaque locations (Multiple correlation analysis, Table 1). Forty-nine (58.3%) patients had BA bending. In 50 patients with dominant VA, the BA was bent contrary to the dominant side in 18 (36%), ipsilateral in 11 (22%) and no bending in 21 (42%). In 34 patients with equivalent VAs, the BA was bent laterally in 20 (58.8%) and not bent in 14 (41.2%). No significant correlation between the plaque locations and the side of BA bending (ventral: *p* = 0.759, dorsal: *p* = 0.765, left lateral: *p* = 0.763, right lateral: *p* = 0.800, Multiple logistic regression analysis).

**Table 1:** Correlation analysis between the difference/ratio of vertebral artery diameters and basilar artery plaque locations

		Ventral wall	Dorsal wall	Lateral wall
Difference				
<0.3 mm	r	−0.292	0.250	−0.018
	p	0.072	0.125	0.914
≥0.3 mm	r	0.076	−0.102	0.138
	p	0.625	0.511	0.366
Ratio				
	r	−0.038	−0.001	−0.010
	*p*	0.731	0.993	0.930

Multiple correlation analysis with p and r-values were given

**Fig. 2:**
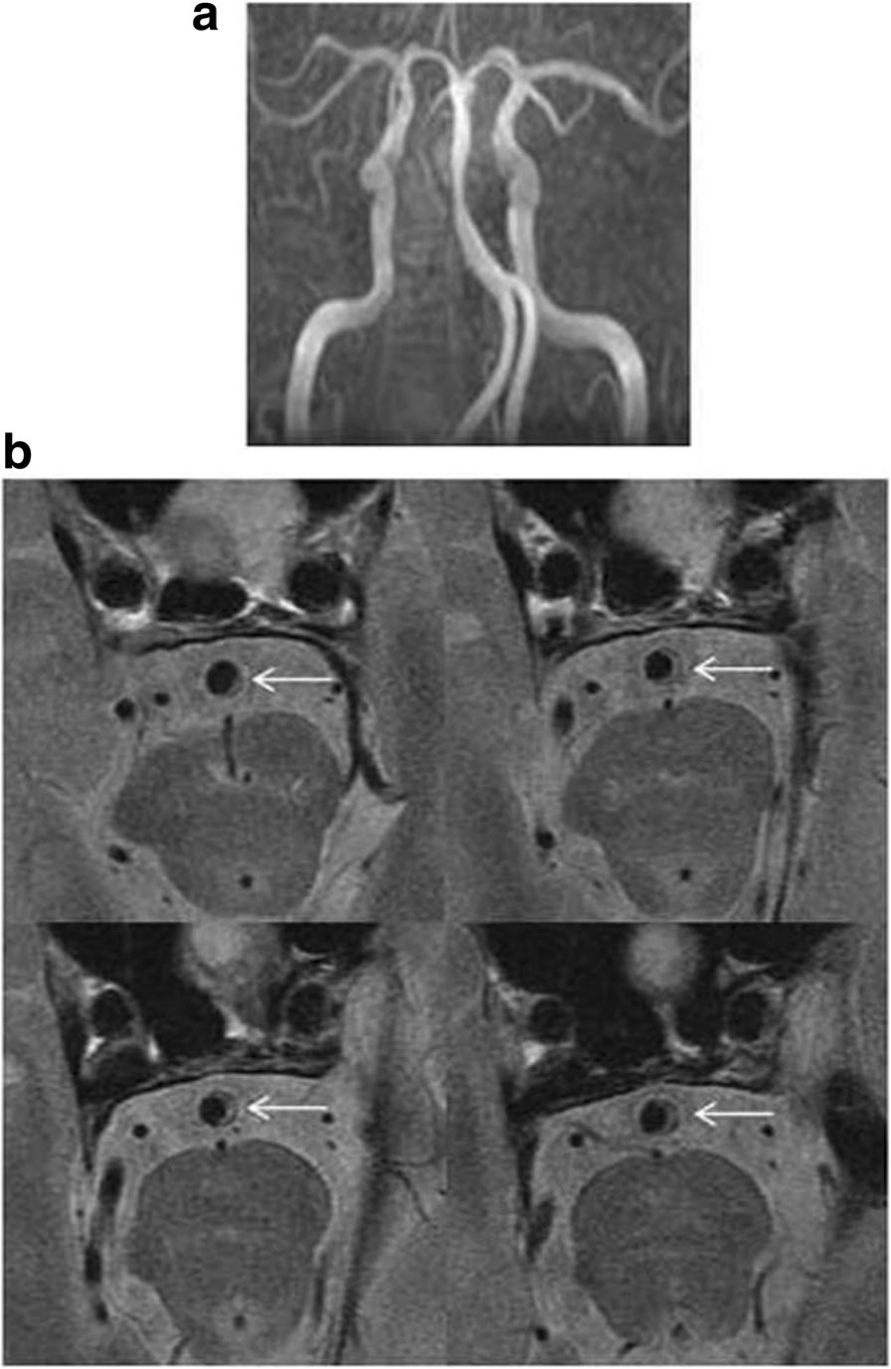
Basilar artery atherosclerosis with Walking configuration. In a basilar artery with Walking configuration (**a**), atherosclerosis involving lateral wall (**b**) can be observed on 4 of 12 consecutive slices on high-resolution magnetic resonance imaging (arrows)

BA plaques were evenly distributed in the vertebrobasilar arteries with Tuning Fork and Dominant-Lambda configurations (Table 2). However, in Hypoplasia-Lambda group, plaques were more frequently located at the dorsal wall (58.57%) than at the ventral(14.43%) and lateral wall (26.71%; *P* = 0.001, Variance analysis and Bonferroni correction test). In Walking group, the plaques more likely occurred at the lateral (49.79%) and dorsal (35.07%) wall than at the ventral wall (14.86%, *P* = 0.02, Fig. 2).

**Table 2:** Geometric configurations and basilar artery plaque locations

Configuration	Ventral wall	Dorsal wall	Lateral wall	*P* *
Walking (14)	14.86%	35.07%	49.79%	0.02^1^
Tuning Fork (16)	32.03%	41.20%	28.90%	0.632
Dominant-Lambda (40)	24.48%	40.03%	35.09%	0.083
Hypoplasia-Lambda (14)	14.43%	58.57%	26.71%	0.001^2^
P	0.268	0.291	0.220	

P indicates comparisons in four groups, P* indicates comparisons in the ventral, dorsal, and lateral sides of BA wall

1, ventral vs. Dorsal, *p* = 0.095; ventral vs. Lateral, *p* = 0.005; dorsal vs. lateral *p* = 0.220

2, ventral vs. Dorsal, *p* = 0.000; ventral vs. Lateral, *p* = 0.284; dorsal vs. lateral *p* = 0.008

## Discussion

In this study, we investigated the correlations between the vertebrobasilar artery geometry and BA plaque locations. It was observed that geometric configurations strongly influenced BA plaque locations. In the vetebrobasilar arteries with Hypoplasia-Lambda and Walking configurations, the BA plaques more frequently occurred at the dorsal or the lateral wall, where the branches of BA mainly arise from. Theoretically, these two configurations may have a higher risk of pontine infarcts due to the mechanism of a parent artery plaque occluding a penetrating artery. The underlying hemodynamic mechanisms, however, remains elusive. Using MRI-Based models, Wake-Buck et al. analyzed the flow dynamics of five healthy subjects in vivo (2 with Walking geometry, 2 with Tuning Fork geometry and 1 with Dominant-Lambda) [11]. The BA geometry strongly influenced both skewing of the velocity profiles and the wall shear stress distributions in the VBS. Unfortunately, the detail of wall shear stress distribution in different geometry configurations was not reported. Another recent work found that the greatest BA-VA angle was observed in patients with posterior plaques and greatest mid-BA angles with lateral BA plaques [13]. We speculate that hemodynamic change occurs at the vertebrobasilar junction and at the mid-BA when the flow bends because of the mid-BA angle. According to our results, the low wall shear stress zones were assumed to be mainly distributed in the dorsal and lateral wall in the Hypoplasia-Lambda and Walking configurations. Further data from computerized hemodynamic studies are required to verify our hypothesis.

No correlation was observed between the ratio/difference of VA diameters and the plaque location. Actually, the asymmetric VAs had been regarded as a congenital variant, that don’t result in a difference in the distribution of shear stress on the BA walls. Ravensbergen et al., by autopsy and a series of junction models, demonstrated that local regions of low wall shear stress (defined as wall shear stress<1 Pa) occurred at the crux of the junction and on the inner wall of the BA curve [14]. The difference of wall shear stress was not related to the VA dominance. On the other hand,similarly, no correlation was observed between the side of BA bending and plaque location. The meandering phenomenon of the BA is also viewed as a long-standing chronic process in adults. Clinical observations have linked these curving arteries to genetic defects, aging, atherosclerosis, hypertension and diabetes mellitus [15]. We consider both asymmetry of VA and BA bending may give a low contribution to the plaque locations.

Our study suffered from several limitations. First, in this study, only 21% patients had a severe stenosis (≥50%). Our results are not appropriate for advanced BA atherosclerosis which is often associated with embolic stroke. Second, patients with any VA stenosis were excluded from the study. Because VA is the upstream vessel of BA, it is not known the hemodynamic effects of VA stenosis to BA atherosclerosis. The hemodynamic roles of VA to BA in our study may be simplified and underestimated. Third, the sample of our study was too small to analyze the relationships between the four configurations and clinical presentations.

## Conclusion

Despite of these limitations, our results may be of clinical importance. In practice, patients with intracranial atherosclerosis may be exposed to similar stroke risk factors but have heterogeneous clinical outcomes. A precise stroke risk stratification for an individual patient is not available. Our results provide a potentially new research direction for primary and secondary prevention of cerebrovascular diseases, that individual intracranial artery geometry should be analyzed in addition to traditional risk factors. Further prospective studies are warranted to testify whether Hypoplasia-Lambda and Walking configurations are independent risk factors for pontine infarcts.

## Additional file

Additional file 1: **Table S1**: Percentage of individual plaque distribution. Calculations of the percentage of individual plaque distribution: The quadrant grouping slice is patient specific. For each stenosis, the percentage of individual plaque distribution was calculated. If a stenosis only had one slice with ventral plaque, for example, the percentage of its individual plaque distribution is ventral 100%, dorsal 0%, lateral 0%. If the stenosis have 3 slices (ventral 2, dorsal 1), the percentage is ventral 66%, dorsal 33%, lateral 0%. (DOCX 18 kb)

## Abbreviations

3D TOF: 3 Dimensional time-of-flight
BA: Basilar artery
FOV: Field of view
HR-MRI: High-resolution magnetic resonance imaging
MRA: Magnetic resonance angiography
PDWI: Proton density weighted imaging
T1WI: T1 weighted imaging
T2WI: T2 weighted imaging
VA: Vertebral artery
VBS: Vertebrobasilar system
