# Supplementary material for: Relationship between the geometry patterns of vertebrobasilar artery and atherosclerosis
Source: BMC Neurol. 2018 Jun 12;18:83. doi: 10.1186/s12883-018-1084-6 (PMC5996488; doi:10.1186/s12883-018-1084-6)
Supplement: Supplementary file 1 — Table S1: Percentage of individual plaque distribution. Calculations of the percentage of individual plaque distribution: The quadrant grouping slice is patient specific. For each stenosis, the percentage of individual plaque distribution was calculated. If a stenosis only had one slice with ventral plaque, for example, the percentage of its individual plaque distribution is ventral 100%, dorsal 0%, lateral 0%. If the stenosis have 3 slices (ventral 2, dorsal 1), the percentage is ventral 66%, dorsal 33%, lateral 0%. (DOCX 18 kb) [file 12883_2018_1084_MOESM1_ESM.docx]

**Supplemental Table 1: Percentage of individual plaque distribution**

|  | Plaque Slices | Ventral(%) | Dorsal(%) | Lateral(%) |
| --- | --- | --- | --- | --- |
| 1 | 7 | 1(14%) | 0 | 6(85%) |
| 2 | 5 | 0 | 2(40%) | 3(60%) |
| 3 | 4 | 0 | 2(50%) | 2(50%) |
| 4 | 1 | 0 | 0 | 1(100%) |
| 5 | 4 | 2(50%) | 0 | 2(50%) |
| 6 | 5 | 2(40%) | 1(20%) | 2(40%) |
| 7 | 8 | 3(37.5%) | 3(37.5%) | 2(25%) |
| 8 | 3 | 0 | 0 | 3(100%) |
| 9 | 4 | 0 | 2(50%) | 2(50%) |
| 10 | 3 | 2(66%) | 1(33%) | 0 |
| 11 | 9 | 1(11%) | 4(44%) | 4(44%) |
| 12 | 6 | 2(33%) | 1(16%) | 3(50%) |
| 13 | 8 | 4(50%) | 0 | 4(50%) |
| 14 | 3 | 0 | 2(67%) | 1(33%) |
| 15 | 4 | 0 | 2(50%) | 2(50%) |
| 16 | 5 | 1(20%) | 1(20%) | 3(60%) |
| 17 | 2 | 0 | 2(100%) | 0 |
| 18 | 4 | 4(100%) | 0 | 0 |
| 19 | 1 | 0 | 1(100%) | 0 |
| 20 | 7 | 1(14%) | 5(71%) | 1(14%) |
| 21 | 5 | 4（80%） | 0 | 1（20%） |
| 22 | 8 | 0 | 4（50%） | 4（50%） |
| 23 | 8 | 3（37.5%） | 2（25%） | 3（37.5%） |
| 24 | 5 | 1（20%） | 3（60%） | 1（20%） |
| 25 | 1 | 0 | 0 | 1（100%） |
| 26 | 11 | 4（36%） | 4（36%） | 3（27%） |
| 27 | 1 | 0 | 0 | 1（100%） |
| 28 | 3 | 2（66%） | 0 | 1（33%） |
| 29 | 1 | 0 | 1（100%） | 0 |
| 30 | 6 | 1（16%） | 4（66%） | 1（16%） |
| 31 | 5 | 0 | 0 | 5（100%） |
| 32 | 7 | 0 | 7（100%） | 0 |
| 33 | 2 | 0 | 0 | 2（100%） |
| 34 | 6 | 1（16%） | 4（66%） | 1（16%） |
| 35 | 9 | 1（11%） | 6(66%) | 2（22%） |
| 36 | 10 | 4(40%) | 2(20%) | 4(40%) |
| 37 | 6 | 3(50%) | 2(33%) | 1（16%） |
| 38 | 3 | 0 | 2(66%) | 1(33%) |
| 39 | 4 | 0 | 3(75%) | 1(25%) |
| 40 | 5 | 1(20%) | 4(80%) | 0 |
| 41 | 4 | 0 | 4(100%) | 0 |
| 42 | 5 | 3(60%) | 1(20%) | 1(20%) |
| 43 | 10 | 0 | 4(40%) | 6(60%) |
| 44 | 12 | 0 | 9(75%) | 3(25%) |
| 45 | 3 | 0 | 2(66%) | 1(33%) |
| 46 | 6 | 0 | 4(66%) | 2(33%) |
| 47 | 6 | 0 | 3(50%) | 3(50%) |
| 48 | 6 | 3(50%) | 0 | 3(50%) |
| 49 | 5 | 0 | 4（80%） | 1(20%) |
| 50 | 3 | 0 | 3（100%） | 0 |
| 51 | 3 | 3(100%) | 0 | 0 |
| 52 | 7 | 5(71%) | 1(14%) | 1(14%) |
| 53 | 4 | 2(50%) | 2(50%) | 0 |
| 54 | 3 | 0 | 2(66%) | 1(33%) |
| 55 | 4 | 2(50%) | 1(25%) | 1(25%) |
| 56 | 5 | 5(100%) | 0 | 0 |
| 57 | 7 | 3(42%) | 0 | 4(57%) |
| 58 | 8 | 7(87.5%) | 0 | 1(12.5%) |
| 59 | 11 | 3（27%） | 3（27%） | 5(45%) |
| 60 | 9 | 2（22%） | 5(55%) | 2（22%） |
| 61 | 7 | 3(42%) | 3(42%) | 1(14%) |
| 62 | 3 | 3(100%) | 0 | 0 |
| 63 | 6 | 0 | 0 | 6(100%) |
| 64 | 6 | 0 | 6(100%) | 0 |
| 65 | 8 | 1(12.5%) | 0 | 7(87.5%) |
| 66 | 4 | 1(25%) | 2(50%) | 1(25%) |
| 67 | 8 | 37.50% | 3(37.5%) | 2(25%) |
| 68 | 8 | 2(25%) | 0 | 6(75%) |
| 69 | 3 | 33% | 66% | 0 |
| 70 | 9 | 0 | 3(100%) | 0 |
| 71 | 3 | 2(66%) | 1(33%) | 0 |
| 72 | 1 | 0 | 1(100%) | 0 |
| 73 | 3 | 0 | 3(100%) | 0 |
| 74 | 5 | 2(40%) | 2(40%) | 1(20%) |
| 75 | 4 |  |  | 4(100%) |
| 76 | 8 | 0 | 6(75%) | 2(25%) |
| 77 | 4 | 0 | 2(50%) | 2(50%) |
| 78 | 1 | 0 | 1(100%) | 0 |
| 79 | 3 | 0 | 2(66%) | 1(33%) |
| 80 | 2 | 0 | 2(100%) | 0 |
| 81 | 3 | 1(33%) | 1(33%) | 1(33%) |
| 82 | 3 | 0 | 3(100%) | 0 |
| 83 | 2 | 0 | 0 | 2(100%) |
| 84 | 2 | 0 | 2(100%) | 0 |
